# Health-Related Social Control and Perceived Stress Among High-Risk Latina Mothers with Type 2 Diabetes and Their At-Risk Adult Daughters

**DOI:** 10.1007/s12529-022-10145-y

**Published:** 2023-01-17

**Authors:** Maribel Cervantes-Ortega, Anton M. Palma, Karen S. Rook, Kelly A. Biegler, Katelyn C. Davis, Emily A. Janio, David B. Kilgore, Emily Dow, Quyen Ngo-Metzger, Dara H. Sorkin

**Affiliations:** 1 Department of Epidemiology, University of California, Irvine, Irvine, CA 92697 USA; 2 Department of Psychological Science, University of California, Irvine, Irvine, CA 92697 USA; 3 Institute for Clinical and Translational Science, University of California, Irvine, Irvine, CA 92697 USA; 4 Department of Medicine, University of California, Irvine, Irvine, CA 92697 USA; 5 Department of Family Medicine, University of California, Irvine, Irvine, CA 92697 USA; 6 Department of Health Systems Science, Kaiser Permanente Bernard J. Tyson School of Medicine, Pasadena, CA 91101 USA

**Keywords:** Health-related social control, Persuasion, Pressure, Perceived stress, Social network

## Abstract

**Background:**

Diabetes-related multi-morbidity and cultural factors place Latinas with diabetes at increased risk for stress, which can threaten illness management. Families provide an ideal focus for interventions that seek to strengthen interpersonal resources for illness management and, in the process, to reduce stress. The current study sought to examine whether participating in a dyadic intervention was associated with reduced perceived stress and, furthermore, whether this association was mediated by persuasion and pressure, two forms of health-related social control.

**Method:**

Latina mothers with diabetes and their at-risk adult daughters participated in either (1) a dyadic intervention that encouraged constructive collaboration to improve health behaviors and reduce stress, or (2) a usual-care minimal control condition. Actor-partner interdependence model analysis was used to estimate the effect of the intervention on dyads’ perceived stress, and mother-daughter ratings of health-related social control as potential mediators.

**Results:**

Results revealed that participating in the intervention was associated with significantly reduced perceived stress for daughters, but not for mothers (β =  − 3.00, *p* = 0.02; β =  − 0.57, *p* = 0.67, respectively). Analyses also indicated that the association between the intervention and perceived stress was mediated by persuasion, such that mothers’ who experienced more health-related persuasion exhibited significantly less post-intervention perceived stress (indirect effect =  − 1.52, 95% CI = [− 3.12, − 0.39]). Pressure exerted by others, however, did not evidence a mediating mechanism for either mothers or daughters.

**Conclusion:**

These findings buttress existing research suggesting that persuasion, or others’ attempts to increase participants’ healthy behaviors in an uncritical way, may be a driving force in reducing perceived stress levels.

## Introduction

Approximately 40% of US adults are expected to develop type 2 diabetes over their lifetimes, and that percentage is even higher for Hispanic men and women—more than 50% [1]. Risk factors for diabetes, such as obesity, poor diet, and physical inactivity, are especially high in the Latino population [1, 2, 3, 4, 5]. Furthermore, individuals of Hispanic descent are 50% more likely to die from diabetes compared to non-Hispanic Whites [1]. Mexican–American women, in particular, have an elevated risk of developing diabetes, as rates of sedentary behavior and obesity or overweight status are high in this group relative to non-Hispanic White women [6].

Managing the day-to-day demands of a chronic disease like diabetes has been shown to be a source of stress [7]. Evidence suggests that people from diverse racial/ethnic backgrounds report more illness-related distress than do their non-Hispanic White counterparts [8]. Moreover, perceived stress has been found to be related to worse diabetes outcomes, including excess body weight [9], poorer glycemic control, long-term complications, and premature mortality [10]. These associations may operate not only through biological mechanisms, such as elevated stress hormones [11, 12], but also through behavioral mechanisms, such as ineffective diabetes self-care behaviors [13]. It warrants noting, as well, that Hispanic men and women also are more likely to experience stressful life events (e.g., financial stressors) and daily hassles (e.g., difficulty paying for medications, poor access to high-quality foods, lack of safe places in which to exercise [14, 15]), which can further increase overall perceived stress and deplete self-regulatory resources that are critical to the self-management of diabetes [16]. Perceived stress constitutes a legitimate intervention target among individuals coping with diabetes [17], and this is likely to be true for Latinas with diabetes who are at risk for elevated perceived stress for multiple reasons [18].

### A Dyadic Approach to Promote Collaboration on Health Behavior Change and Stress Reduction

Many of the serious complications of type 2 diabetes can be prevented or delayed by engaging in healthy lifestyle behaviors, such as eating an appropriate diet, controlling one’s weight, and managing stress; thus, healthy behavior interventions may be effective in preventing diabetes and/or minimizing its downstream consequences. Interventions focused on improving health outcomes for individuals with diabetes have increasingly focused on dyadic interventions that include the participation of a family member [19, 20, 21] as family members are in a unique position to monitor and seek to influence the individual's with diabetes health behavior. This trend marks a departure from earlier approaches that focused largely on changing an individual’s behavior [22]. Less common among the newer dyadic approaches, however, are interventions that pair family members who share the same risks for diabetes or its complications as well as similar needs to modify unhealthy behaviors [23]. This novel intervention approach is particularly well suited to at-risk Latinas given the central role of the family in Hispanic culture.

Hispanic family members often share beliefs and opinions around health issues, thereby mutually influencing each other’s attitudes toward health behaviors and, among those with type 2 diabetes, disease management [16, 24]. In fact, family dynamics and expectations are among the strongest influences on the management and treatment of diabetes in Hispanics [25]. Additionally, daughters often learn about the preparation of traditional foods from their mothers and, as a result, may acquire beliefs and practices relevant to obesity risks [26, 27]. As stress has been shown to be an important factor for undermining engagement and weight loss outcomes, especially among low-income individuals, efforts to promote stress reduction are often incorporated into weight loss interventions [28]. Culturally-tailored interventions for Latinas that foster health-related collaboration and stress reduction between mothers and daughters who share similar health risks have the potential to yield substantial lifestyle changes [23] and, perhaps, to reduce stress that might otherwise interfere with lasting lifestyle changes.

One such study sought to evaluate the feasibility of a lifestyle intervention, Unidas por la Vida (United for Life), in which Mexican–American mothers who had type 2 diabetes and their overweight/obese adult daughters collaborated in an effort to change shared health behaviors [23]. Results revealed that mother-daughter dyads who participated in the intervention lost significantly more weight than did control dyads who received usual care alone. Furthermore, intervention dyads were also more likely to report eating foods with lower glycemic load and less saturated fat by the end of the 16-week intervention period compared to their counterparts [23]. This study, however, did not examine the impact of the intervention on stress processes.

### The Potential Role of Health-Related Social Control in Health Behavior Change and Perceived Stress

When unhealthy behaviors put people at risk for serious illness, their close social network members often intervene to try to prompt health behavior change. Such efforts to influence or regulate another person’s health behaviors have been termed health-related social control [29, 30]. For example, meals are often consumed with social network members who are in a position to monitor and comment on a person’s dietary intake and/or weight-loss efforts [31, 32, 33]. Such scrutiny is typically intended to influence recipients to increase healthy behaviors and decrease their unhealthy behaviors [34].

The effects of social control attempts may depend, in part, on the types of strategies used by social network members. Efforts to prompt or persuade another person to improve his or her health behaviors, termed persuasive social control, have been found to elicit positive health behavior change and positive emotional responses in some studies [35, 36, 37, 38] but not others [39, 40]. In the context of chronic illness, for example, family members who engage in persuasive social control efforts may be perceived as allies in the challenge of managing illnesses [41]. Having an ally, especially someone who needs to make similar health behavior changes, might make the task of managing an illness less daunting, thereby reducing perceived stress.

Alternatively, some forms of influence, however well-intentioned, can amplify rather than reduce stress. Although social control efforts by network members are often intended to promote improved health behavior, recipients may not always welcome others’ efforts. Social control strategies that involve pressure, such as criticizing or expressing doubts about the recipient’s health behaviors, have often been found to be ineffective or even counterproductive in changing behavior. For example, a study that investigated the effects of negative social control (defined as pressure or scolding) on physical activity in healthy couples found that participants engaged in less moderate-to-vigorous physical activity when their partners had exerted more negative control over a 7-week period [42]. Furthermore, social control attempts, particularly those that involve pressure, may communicate that an individual has poor self-control, which could erode feelings of self-efficacy, trigger resentment, and perhaps contribute to relationship tensions. For example, negative social control was linked to reactance, an aversive motivational state that arises when an individual perceives their behavioral freedoms have been threatened or lost, and negative affect, suggesting that it may be that these kinds of social control attempts that contribute to feelings of stress [43].

### The Current Study

The current study sought to add to the literature by examining the extent to which health-related social control may be associated with perceived stress, thus adding to a body of research that has focused primarily on health behavior as an outcome. This question was examined in the context of a 16-week intervention (Unidas por la Vida) in which mothers and adult daughters at risk for developing, or experiencing complications of, type 2 diabetes collaborated together to improve their health behavior and to reduce their perceived stress compared to other participants who were randomly assigned to a usual-care minimal control condition. The study also sought to examine whether the potential role of the intervention in reducing in perceived stress was mediated by persuasion and pressure, two forms of health-related social control, as the intervention [23] included a focus on not only improving health behavior but also reducing stress in the women’s lives by encouraging positive health-related interpersonal exchanges and discouraging critical or nagging health-related interpersonal exchanges. Thus, the goals of the current study were to examine (1) whether participating in the Unidas intervention was associated with a reduction in perceived stress over the 16-week study period; (2) whether persuasion and pressure, two forms of health-related social control, mediated this association; and (3) whether the effects of health-related social control were driven by the individual or her dyad partner. To address the latter question, we employed the actor-partner interdependence model (APIM), which unpacks the mutual influence of dyadic partners by modeling the mother and her adult daughter as nested within the dyad [44]. APIM allows the simultaneous estimation of the effect, within the dyad, that each individual has on herself (actor effect) and on the other person (partner effect). Taking both the mother and her adult daughter into account can inform the design of complex, behavioral health interventions by enriching our understanding of the dyadic influence on perceived stress as an outcome. To our knowledge, this study is among the first to assess the mediating effects of persuasion and pressure in a dyadic weight-loss intervention with perceived stress as the primary outcome. We hypothesized that (1) participation in the intervention would be associated with a reduction in perceived stress; (2) participants’ reports of health-related persuasion, but not health-related pressure, would mediate this association; and (3) mothers’ and daughters’ reports of health-related persuasion would be associated with both their own and their partners’ reduction in perceived stress, whereas their reports of health-related pressure were expected to be associated with an increase in their own and their partners’ perceived stress.

## Method

### Participants

Women identified through patient registries from two Federally Qualified Health Centers (clinics that receive federal funding to provide health care to low income, underinsured, and underserved populations) as being Hispanic, having a diagnosis of type 2 diabetes, and having a body mass index (BMI) of > 25 were contacted by phone to assess initial study interest and eligibility. Women who showed interest in participating completed an informed consent form and a Health Insurance Portability and Accountability Act (HIPAA) waiver form, allowing the study team access to the participant’s medical record. Subsequently, with the participant’s permission, study personnel contacted the participant’s daughter, and if she was interested, obtained her informed consent and HIPAA waiver authorization access. All forms were available in both English and Spanish. All study conducts and procedures were approved by the University of California, Irvine Institutional Review Board (#2009-7225).

Of the 882 dyads who were assessed for eligibility, 323 dyads met the eligibility criteria to participate in the study. Women were recruited based on the following criteria: (1) self-identified as Latina, (2) had a daughter over the age of 18, (3) BMI ≥ 25, (4) ICD-9 diagnosis of type 2 diabetes (mothers only), (5) mothers and daughters independently consented to participate, and (6) lived within 25 miles of each other [45]. Women were excluded if they were pregnant or became pregnant during the course of the study, were unable to provide informed consent, or had contraindications for engaging in moderate physical activity. Of the 323 eligible dyads, 218 had one member who declined to participate, 10 had contraindications to engage in a weight loss intervention upon physician assessment, and 6 declined to participate following physician clearance. Thus, the final study sample for this randomized control trial consisted of 89 dyads (178 women) [23, 45]. The attrition rate over the course of the study was low (3.9%), as only four women withdrew from the intervention group (1 mother-daughter dyad and 2 individual daughters) and three women withdrew from the Usual Care Control group (1 mother-daughter dyad and 1 mother).

### Dyadic Intervention

The Unidas por la Vida dyadic intervention was modeled after the Diabetes Prevention Program (DPP) Lifestyle change program [46]. Although the DPP has been effectively implemented in the Latino population, the Unidas por la Vida program was further adapted to capitalize on local, public, community-based resources (e.g., partnership with local community college to use outdoor physical activity spaces) in an effort to support sustained behavior change, and to promote a collaborative partnership between mother and daughter to achieve shared weight loss goals [23].

The 16-week intervention consisted of four group classes, eight home visits, and four booster phone calls. The curriculum featured common strategies employed in weight loss programs, including (1) setting weight loss goals, (2) meal planning and preparation, (3) identifying obstacles and barriers, (4) problem-solving, (5) getting back on track after experiencing setbacks, and (6) managing stress. In participating in the Unidas intervention together, mother/daughter dyads were encouraged to engage in each of these weight loss strategies together, including meal planning and exercising, frequently checking in with each other to support meeting weekly weight loss and healthy lifestyle goals, and working together to identify and overcome individual and dyadic barriers, retain motivation, and manage stress. Furthermore, in the less formally structured interactions with participants, the topic of stress often arose, and the Lifestyle Community Coaches then engaged with women in problem-solving discussions of ways to manage stress.

In addition to formal elements of the intervention that focused on stress management, it is plausible that having a family member with whom to collaborate on reducing shared health risks could also help to reduce stress. Thus, in addition to the explicit educational focus on stress reduction, the dyadic nature of the intervention itself (which encouraged women to function as allies in achieving shared health goals), as compared with usual care, could play a role in reducing stress.

### Usual Care

Participants randomized to the Usual Care control group also completed baseline and 16-week assessments. In addition, mothers (with diagnosed diabetes) received National Diabetes Education Program materials that were mailed to their homes. Daughters also received mailed diabetes prevention materials that discussed diabetes risk factors and lifestyle factors known to prevent diabetes, developed by the National Institute of Diabetes and Digestive and Kidney Diseases. All participants were advised to continue usual care with their primary care provider.

### Measures

Demographic information, health status, perceived stress, and measures of health-related social control (persuasion and pressure) were assessed through self-reported questionnaires at baseline and 16 weeks post-intervention. All surveys were available to participants in both English and Spanish.

## Perceived Stress

Perceived stress was measured using the 10-item Perceived Stress Scale [47]. This measure assesses perceptions of ongoing life stress and is widely used in populations with chronic conditions. Items included questions such as “How often have you felt you were unable to control the important things in your life?” and “How often have you felt difficulties were piling up so high that you could not overcome them?” Item responses ranged from 0 = never to 4 = very often, and the sum of ratings on all 10 items was computed as the total scale score. The scale showed good reliability in the current sample (Cronbach’s α baseline = 0.82 and 16 weeks post-intervention = 0.81).

## Health-Related Social Control

Two forms of health-related social control were assessed. *Persuasion* was assessed using three items, such as “Over the past month, how often did the important people in your life try to do something to get you to improve your food choices or exercise regimen?” and “Over the past month, how often did the important people in your life try to persuade you to do more to follow your diet or exercise regimen?” (0 = not at all to 6 = every day) [37, 40]. The composite measure, computed as a mean of the three items, demonstrated strong reliability in this sample (Cronbach’s α Baseline = 0.93 and 16 weeks post-intervention = 0.92). *Pressure* was assessed using four items, such as “Over the past month, how often did the important people in your life criticize your poor food choices or lack of physical activity?” and “Over the past month, how often did the important people in your life question or express doubts about your poor food choices or physical inactivity?” (0 = not at all to 6 = every day) [40, 48]. The composite measure, computed as a mean of the four items, showed strong reliability in the current sample (Cronbach’s α baseline = 0.89 and 16 weeks post-intervention = 0.85).

### Statistical Analysis

The goal of the present study was to test whether or not participation in the intervention was associated with a reduction in perceived stress over a 16-week time period and whether health-related social control mediated the effect of the intervention on perceived stress. To examine these associations, we fit a structural equation model (SEM) to estimate direct effects of the Unidas por la Vida Intervention vs. Usual Care on perceived stress and indirect effects through persuasion and pressure estimated in parallel. Separate paths were created for mothers and daughters in the model, following the appropriate APIM design for distinguishable dyads to enable the assessment of each member’s individual influence on the other [49]. Analyses controlled for mothers’ and daughters’ co-resident status (no = 0, yes = 1), baseline perceived stress, and baseline social control [23]. Variances were estimated via bootstrapping and models were fit using full information maximum likelihood to account for missing data [50]. Mediation results were interpreted using the framework developed by Zhao et al. [51]. This innovative framework removes the necessity of a significant direct effect when the mediator is not controlled for, as is required by the Baron and Kenny approach [52].

## Results

### Initial Analyses

We first examined demographic characteristics for mothers and daughters and conducted *t*-tests to assess whether there were significant changes in persuasion, pressure, and perceived stress from baseline (T1) to follow-up (T2). Of the 89 dyads in the study, 88 completed baseline and post-intervention surveys and were included in the analysis. Of those 88 dyads, 51 were in the intervention group and 37 were in the control group. A majority of the mothers (72.9%) lived with their adult daughters who were participating in the study. Overall, 97.7% of the mothers and 67.5% of the daughters were born outside of the USA, with 72.3% of the mothers and 10.8% of the daughters speaking Spanish only. Although 39.2% of mothers and 55.6% of daughters reported either full- or part-time work, many participants were low income, with 41.5% of mothers and 55.7% of daughters reporting a yearly income below $15,000. Additional details about the study population can be found elsewhere [23, 45, 53].

Analyses revealed that, for both mothers and daughters, perceived stress decreased over the 16-week period (ΔMean_T2-T1_: mothers =  − 2.1, *p* = 0.05; daughters =  − 2.1, *p* = 0.03). Persuasion also increased from baseline to post-intervention for both members of the dyad (ΔMean_T2-T1_: mothers = 0.6, *p* = 0.06; daughters = 0.7, *p* = 0.004). Pressure, in contrast, did not change over the 16-week period for either mothers or daughters (ΔMean_T2-T1_: mothers =  − 0.1, *p* = 0.66; daughters = 0.1, *p* = 0.57).

### Direct and Indirect Dyadic Effects on Perceived Stress

**Fig. 1:**
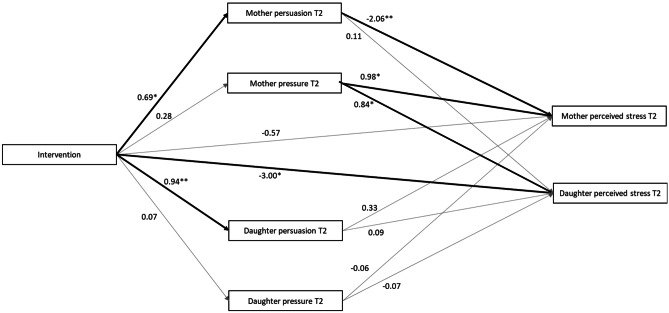
Unstandardized (beta) coefficients from the SEM analysis testing for actor-partner effects on the direct and indirect relation of intervention to T2 perceived stress. T2 = Time 2. Bold indicates significant path between variables. *p < 0.05, **p < 0.001

Results from direct paths in the structural equation model are shown in Table 1. Overall, the model had a goodness of fit of 0.87, indicating a good fit, even though the RMSEA value was 0.15. The direct effect of the intervention on T2 perceived stress differed between mothers and daughters such that there was a significant decrease in stress for daughters (β =  − 3.00, *p* = 0.02), but not for mothers (β =  − 0.57, *p* = 0.67). Participating in the intervention was also associated with a significant increase in mothers’ and daughters’ reports of experiencing persuasion over the 16-week period (β = 0.69, *p* = 0.01; and β = 0.94, *p* = 0.002, respectively). The intervention, however, did not have a significant effect on T2 pressure. Higher levels of persuasion reported by mothers across the 16-week period was associated with a significant decrease in their own T2 perceived stress (β = -2.06, *p* < 0.001), but not daughters’ stress. In contrast, higher levels of pressure reported by mothers was associated with a significant increase in T2 stress for both mothers and daughters (β = 0.98, *p* = 0.02; and β = 0.84, *p* = 0.03, respectively). These findings suggest that mothers’ reports of pressure impact both their own stress and their daughters’ stress, whereas reports of persuasion only impact their own stress. Daughters’ reports of persuasion and pressure over the 16-week period, on the other hand, did not have any significant impact on themselves or their mothers’ perceived stress. All significant paths in the SEM model are shown in Fig. 1.

**Table 1:** Direct effects of the intervention and components of health-related social control (persuasion, pressure) on follow-up (T2) perceived stress

	Mothers			Daughters		
Direct effects of:	β	SE	*p*-value	β	SE	*p*-value
Intervention on persuasion	**0.69**	**0.27**	**0.01**	**0.94**	**0.28**	**0.002**
Intervention on pressure	0.28	0.33	0.40	0.07	0.33	0.82
Intervention on stress	− 0.57	1.32	0.67	− **3.00**	**1.22**	**0.02**
Mothers' persuasion on stress	− **2.06**	**0.49**	** < 0.001**	0.11	0.48	0.83
Daughters' persuasion on stress	0.33	0.56	0.55	0.09	0.58	0.87
Mothers' pressure on stress	**0.98**	**0.41**	**0.02**	**0.84**	**0.38**	**0.03**
Daughters' pressure on stress	− 0.06	0.47	0.89	− 0.07	0.44	0.86

Bold indicates significant effect

The covariate-adjusted beta estimates and bootstrapped 95% confidence intervals for the direct and indirect effects of the intervention on T2 perceived stress for mothers and daughters are reported in Table 2. Total effect (TE) estimates indicate that the intervention was associated with an overall reduction in perceived stress from T1 to T2, although the reduction is larger in magnitude for daughters than it is for mothers (TE =  − 2.61, [− 4.73, − 0.59]; and TE =  − 1.41, [− 3.57, 1.04], respectively). The direct effect (DE) of the intervention on T2 perceived stress similarly showed a significant decrease in stress for daughters (DE =  − 3.00, [− 5.04, − 0.70]), but not for mothers (DE =  − 0.57, [− 3.59, 2.72]). We did not observe significant total indirect effects (IE) for mothers or daughters (IE =  − 0.84, [− 2.52, 0.75] for mothers and IE = 0.39 [− 1.18, 1.30] for daughters). However, we did observe significant indirect effects of the intervention through persuasion reported by mothers on their own stress levels (IE =  − 1.42 [− 2.63, − 0.38]). Neither persuasion reported from daughters nor reported pressure from mothers or daughters showed significant mediating effects for either mothers’ or daughters’ stress on average. These collective findings indicate that mediation occurred for mothers, but not for daughters.

**Table 2:** Mediating effects for the actor-partner interdependence mediation model with intervention as the independent variable, T2 mothers’ and daughters’ persuasion and pressure as mediators, and T2 mothers’ and daughters’ perceived stress as dependent variables

	**Effect on mothers’ stress**			**Effect on daughters’ stress**		
	Estimate	95% CI		Estimate	95% CI	
Total effect	− 1.41	− 3.57	1.04	− **2.61**	− **4.73**	− **0.59**
Direct effect	− 0.57	− 3.59	2.72	− **3.00**	− **5.04**	− **0.07**
Total indirect effect	− 0.84	− 2.52	0.75	0.39	− 1.18	1.30
Indirect effects through:						
Mothers' persuasion	− **1.42**	− **2.63**	− **0.38**	0.07	− 0.43	0.67
Daughters' persuasion	0.31	− 0.86	1.32	0.09	− 0.95	0.93
Mothers' pressure	0.27	− 0.35	0.98	0.23	0.33	0.81
Daughters' pressure	0.00	− 0.34	0.30	− 0.01	− 0.33	0.19

95% confidence intervals represent bootstrapped values after 5,000 iterations

Bold indicates significant effect

## Discussion

Health researchers have become increasingly aware that for people with type 2 diabetes, complications may develop as a result of increased stress and negative coping strategies [54, 55, 56, 57, 58]. To develop more effective programs for individuals with type 2 diabetes, newer intervention models similar to Unidas por la Vida have begun to incorporate family members who share similar health risks [59]. Building upon the well-documented importance of the family in Hispanic culture, the Unidas program targeted dyadic relationships between mothers with type 2 diabetes and their obese/overweight daughters in order to help them work collaboratively to maintain healthy lifestyles and ultimately reduce stress. The current study examined whether participating in the Unidas intervention was associated with a reduction in perceived stress and to further examine the extent to which persuasion and pressure, two forms of health-related social control, mediated this association.

Our findings revealed that participating in the Unidas por la Vida weight loss intervention was associated with significantly reduced perceived stress for daughters, but not for mothers. Moreover, increased persuasion from T1 to T2 was associated with significantly less perceived stress among mothers but not daughters. This difference may arise, in part, from differences between the mothers’ and daughters’ overall health status. Upon recruitment, mothers were already diagnosed with type 2 diabetes whereas daughters were at risk of developing the disease. Mothers may have felt a greater sense of urgency about managing their illness and making needed health-behavior changes. Therefore, persuasion from others to manage their health may have helped the mothers feel relieved that others were monitoring their health behavior and encouraging them to stay on track. Daughters, on the other hand, may not have felt the same sense of urgency about their health status and, as a result, may not have experienced a sense of allegiance or relief when others suggested that they change their health behavior [60].

In contrast, mothers’ increased reports of pressure from T1 to T2 was associated with increased stress for both mothers and daughters. Although health-related pressure, like persuasion, is intended to protect a person’s health by advocating sound health behaviors, pressure may come at an emotional cost [61]. Research has alluded to a dual effect, where social control may help reduce the occurrence of poor health behaviors while also arousing psychological distress and, in the current study, perceived stress [35, 62]. In the context of a weight loss intervention, findings from the current study buttress existing research by suggesting that health-related pressure may invoke feelings of irritation or frustration in the recipient and may also convey that the recipient is doing a poor job of managing her illness [61]. Therefore, the inefficacy that pressure may convey could add to, rather than decrease, perceived stress among individuals who are contending with a chronic illness.

These patterns of reduced stress, according to Zhao et al. indicate mediation for mothers but not for daughters [51]. Looking specifically at the indirect paths, a mediating effect on post-intervention perceived stress is evident through mothers' health-related persuasion. No effect, however, remains for the paths through pressure. These findings are also consistent with the literature suggesting that persuasion is the driving force in eliciting change [34, 37, 63, 64]. Persuasion, the method by which members attempt to increase participants’ health behaviors in a nonjudgmental way, may be interpreted as allies trying to encourage positive health behaviors which seem to have a stronger impact in generating change [65]. The results of the current study echo those of earlier studies in suggesting that persuasive and pressure strategies of social control have distinctive effects that warrant differentiation in studies of individuals who have or are at risk for chronic illness [36, 37, 63, 64].

Although this study is among the first to assess the mediating effects of health-related persuasion and pressure in a dyadic weight-loss intervention with perceived stress as the primary outcome, several study limitations exist. First, the data were collected at only two time points that spanned a 16-week period. Although two time points allow for the assessment of temporal changes, our study design did not allow us to capture additional fluctuations in feelings of perceived stress that may have occurred during or long after the intervention. Two time points, however, improve upon cross-sectional designs for mediation analyses because of their ability to reduce variations in direction and magnitude [66, 67]. Additionally, having more than a single time point reduces the likelihood of finding support for mediation effects when there is no true mediation occurring [65]. A second limitation is that the study did not include an assessment of diabetes-related stressors, such as a measured of perceived diabetes stress or a checklist of diabetes stressors. It is possible that such a diabetes-specific measure of stress might have yielded more evidence of the effects of the intervention or health-related social control for both mothers and daughters. In a related vein, it is possible that unknown mediators may have been omitted from the analysis. For example, coping strategies might have mediated the effects of the intervention on daughters’ stress, and future studies might benefit from including this and other mediators. Third, our model did not exhibit adequate goodness of fit on all fit indices. It is possible that this is driven mostly by small sample size, as RMSEA is known to be inflated in situations with few observations. Care should be taken in drawing conclusions from the model’s results, and our findings would need to be replicated in larger studies. Lastly, it is unclear whether the study findings are generalizable to groups outside of the Hispanic culture, males, or those of a higher socio-economic status.

Our study highlights the importance of examining social network processes as they may affect health behaviors and perceived stress among individuals who have or are at risk for chronic illness. Findings highlight, for Hispanic women in particular, the importance of familial relationships where multiple members share similar health risks for stress and the potential complications that may develop as a result. Increasing awareness of the potential benefits of persuasion through social networks is especially important among those who seek to aid individuals navigating important health behavior changes, including family members and health care providers alike. Encouraging persuasive exchanges across individuals has the potential to increase long term health and well-being, especially for those managing a chronic illness.

## Data Availability

The data that support the findings of this study may be available on request from the corresponding author, DS. The data are not publicly available due to their containing information that could compromise the privacy of research participants.
